# A multi-center analysis of single-fraction versus hypofractionated stereotactic radiosurgery for the treatment of brain metastasis

**DOI:** 10.1186/s13014-020-01522-6

**Published:** 2020-05-28

**Authors:** Jill S. Remick, Emily Kowalski, Rahul Khairnar, Kai Sun, Emily Morse, Hua-Ren R. Cherng, Yannick Poirier, Narottam Lamichhane, Stewart J. Becker, Shifeng Chen, Akshar N. Patel, Young Kwok, Elizabeth Nichols, Pranshu Mohindra, Graeme F. Woodworth, William F. Regine, Mark V. Mishra

**Affiliations:** 1 Department of Radiation Oncology, University of Maryland Medical Center, Baltimore, MD USA; 2 Department of Pharmaceutical Health Services Research, University of Maryland School of Pharmacy, Baltimore, MD USA; 3 Department of Radiation Oncology, University of Maryland School of Medicine, Baltimore, MD USA; 4 Chesapeake Oncology Hematology Associates, Glen Bernie, MD USA; 5 Department of Neurosurgery, University of Maryland School of Medicine, Baltimore, MD USA

**Keywords:** Brain metastasis, Stereotactic radiosurgery, Hypofractionated stereotactic radiosurgery, GammaKnife, Normal tissue injury, Biologic effective dose

## Abstract

**Background:**

Hypofractionated-SRS (HF-SRS) may allow for improved local control and a reduced risk of radiation necrosis compared to single-fraction-SRS (SF-SRS). However, data comparing these two treatment approaches are limited. The purpose of this study was to compare clinical outcomes between SF-SRS versus HF-SRS across our multi-center academic network.

**Methods:**

Patients treated with SF-SRS or HF-SRS for brain metastasis from 2013 to 2018 across 5 radiation oncology centers were retrospectively reviewed. SF-SRS dosing was standardized, whereas HF-SRS dosing regimens were variable. The co-primary endpoints of local control and radiation necrosis were estimated using the Kaplan Meier method. Multivariate analysis using Cox proportional hazards modeling was performed to evaluate the impact of select independent variables on the outcomes of interest. Propensity score adjustments were used to reduce the effects confounding variables. To assess dose response for HF-SRS, Biologic Effective Dose (BED) assuming an α/β of 10 (BED_10_) was used as a surrogate for total dose.

**Results:**

One-hundred and fifty six patients with 335 brain metastasis treated with SF-SRS (*n* = 222 lesions) or HF-SRS (*n* = 113 lesions) were included. Prior whole brain radiation was given in 33% (*n* = 74) and 34% (*n* = 38) of lesions treated with SF-SRS and HF-SRS, respectively (*p* = 0.30). After a median follow up time of 12 months in each cohort, the adjusted 1-year rate of local control and incidence of radiation necrosis was 91% (95% CI 86–96%) and 85% (95% CI 75–95%) (*p* = 0.26) and 10% (95% CI 5–15%) and 7% (95% CI 0.1–14%) (*p* = 0.73) for SF-SRS and HF-SRS, respectively. For lesions > 2 cm, the adjusted 1 year local control was 97% (95% CI 84–100%) for SF-SRS and 64% (95% CI 43–85%) for HF-SRS (*p* = 0.06). On multivariate analysis, SRS fractionation was not associated with local control and only size ≤2 cm was associated with a decreased risk of developing radiation necrosis (HR 0.21; 95% CI 0.07–0.58, *p* < 0.01). For HF-SRS, 1 year local control was 100% for lesions treated with a BED_10_ ≥ 50 compared to 77% (95% CI 65–88%) for lesions that received a BED_10_ < 50 (*p* = 0.09).

**Conclusions:**

In this comparison study of dose fractionation for the treatment of brain metastases, there was no difference in local control or radiation necrosis between HF-SRS and SF-SRS. For HF-SRS, a BED_10_ ≥ 50 may improve local control.

## Background

Radiotherapy for the treatment of brain metastases has evolved significantly over the past several decades. Whole brain radiation therapy (WBRT) has historically been used after surgical resection to improve locoregional control [1]. Stereotactic radiosurgery (SRS) was later established as a feasible and safe treatment option for recurrent brain tumors in the setting of prior brain radiotherapy [2]. Subsequent randomized trials further supported the use of SRS alone in the definitive setting after demonstrating no difference in overall survival but less neurocognitive decline compared to combination treatment with WBRT [3–6]. Similar findings were also observed in the post-operative setting [7, 8] resulting in SRS becoming the standard radiation approach for treating brain metastases. The ability to treat multiple brain metastases simultaneously has further broadened the scope of SRS in this patient population [9–11].

Target lesion size and proximity to critical structures are the main limiting factors of single-fraction stereotactic radiosurgery (SF-SRS). Several studies have directly correlated tumor size and exposure of normal brain to higher doses of radiation with an increased risk of radiation necrosis as well as worsened local control [2, 12–15]. When treating larger lesions, a hypo-fractionated stereotactic approach has been increasingly utilized in attempt to improve local control while also minimizing the risk of toxicity to normal tissue [16–24]. In addition to tumor-specific considerations, the ability to deliver SF-SRS may also be limited by the availability of technology. This is due to both the cost needed to upgrade existing machines to be SF-SRS-compatible and the additional resources that are required to comply with more robust quality-assurance programs [25–27].

SF-SRS versus hypo-fractionated stereotactic radiosurgery (HF-SRS) has been compared retrospectively, revealing similar to improved local control with decreased treatment-related toxicity associated with HF-SRS [28–30]. However, the optimal HF-SRS dosing regimen is unclear and data evaluating dose-response to different fractionated regimens are limited. Here, we compare our experience within a multi-center academic network using SF-SRS and HF-SRS to treat brain metastases utilizing various HF-SRS dosing regimens. Unique to our study is an analysis of a dose response by using biological effective dose information (BED) as a surrogate measure for total dose.

## Materials and methods

This retrospective study included patients treated across 5 radiation oncology centers associated with a single academic institution. Inclusion criteria were age ≥ 18 years, a pathologically confirmed systemic malignancy and an MRI confirming the presence of brain metastasis. Patients with prior surgical, systemic and/or radiation (including prior WBRT and/or SRS to other lesions) treatment for brain metastases were included. Lesions without adequate follow up information, defined as no brain MRI ≥ 28 days after SRS and/or lack of sufficient follow up information to discern treatment-related toxicity, were excluded (*n* = 62). Lesions re-irradiated with SRS for local failure were also excluded. Corticosteroids were administered to patients with neurologic symptoms at presentation and to asymptomatic patients at the discretion of the treating physician if there was concern for neurologic sequelae from post-treatment swelling (i.e. based on size, location, degree of perilesional edema). Clinical, radiographic and pathologic characteristics for each individual lesion were collected through retrospective chart review. All data was recorded via a web-based software program (REDCap®) to ensure data entry was consistent and protected. There were 10 patients who received both SF- and HF-SRS to different lesions, with SF-SRS followed by HF-SRS to new lesions being the most common order of treatment (*n* = 6). Patient characteristics (i.e. age, gender, KPS, etc.) were reported at the time of their first SRS treatment.

### RT planning and treatment techniques

All patients were simulated in the supine position. For SF-SRS, patients were immobilized using a stereotactic frame (Gamma Knife) or the Encompass immobilization system (QFix, Avondale, PA USA). For HF-SRS, a standard thermoplast head and neck mask was used for immobilization. The GTV was defined as the contrast enhancing tumor on T1 contrast enhanced thin-sliced (1 mm) axial MRI fused with a treatment planning CT scan (1 mm slice thickness). Per institutional guidelines, no additional margin was added for clinical tumor volume (CTV) in the definitive treatment setting. For post-operative treatment, a CTV was generated by adding a 2 mm margin to the post-op cavity including any residual enhancement [31]. For patients treated using frameless linac-based SF-SRS or HF-SRS, a PTV margin was added at the discretion of treating physician. There was no PTV margin used for lesions treated with GK.

SF-SRS was delivered with either Gamma Knife (Elekta, Stockholm, Sweden) (GK) (*n* = 148) or frameless linac-based SRS on the Varian Edge linear accelerator (Varian, Palo Alto, CA USA) (*n* = 74). The prescription dose was prescribed to the 50% isodose line (IDL) for GK and 80% IDL for frameless linac-based SF-SRS. The prescription dose for SF-SRS was as follows: tumors ≤2 cm, 2-3 cm and 3–4 cm were treated to 20–24 Gy, 18 Gy and 15 Gy, respectively. The dose was reduced to 20Gy for lesions ≤2 cm in the setting of planned prior WBRT, as per previous data suggesting no benefit of dose escalation above 20 Gy [32]. There was no dose reduction for larger (2-4 cm) tumors. For post-operative SF-SRS (*n* = 11), prescription dose was determined by surgical cavity volume. For HF-SRS, the prescribed dose regimens were as follows: 10Gy × 3 (*n* = 4), 9Gy × 3 (*n* = 13), 8Gy × 3 (*n* = 24), 7Gy × 3 (*n* = 29), 6Gy × 3 (*n* = 5), 5Gy × 3 (*n* = 5), 6Gy × 5 (n = 13), 5–5.5Gy × 5 (*n* = 19) and 8Gy × 2 (n = 1). For linac-based SF-SRS, the amount of normal brain tissue receiving 12 Gy was limited to < 10 cc as a primary constraint and < 20 cc as a secondary constraint per institutional guidelines. There were no pre-specified normal brain tissue constraints for HF-SRS during the time period of this study. The biological effective dose (BED) based on the linear quadratic model of irradiated cell survival was calculated using an estimated α/β ratio of 10 (BED_10_) [33].

### Treatment delivery

GK treatment delivery was based on a single stereotactic coordinate system. In the case of frameless linac radiation delivery, a cone-beam cat-scan (CBCT) was used to localize the tumor, and the PerfectPitch (Varian, Palo Alto, CA USA) robotic couch capable of six degree of freedom motion was used to replicate patient position during treatment simulation. The optical surface monitoring system (OSMS, Varian, Palo Alto CA USA) was used to monitor intra-fraction patient motion during radiation delivery.

HF-SRS was performed using either the Trilogy or Truebeam linac (Varian, Palo Alto, CA, USA) with daily CBCT and 4-degree of freedom motion couch or on the Varian Edge machine. There was no optical surface imaging for intra-fraction monitoring during treatment on the Trilogy or Truebeam linacs.

### Follow up

Patients were seen in follow-up 4–6 weeks after completing radiation treatment and every 3 months thereafter. A follow-up MRI was obtained at 4–6 weeks and then at an average of 3 month intervals. The presence of local failure and radiation necrosis were determined based on pathology and clinical/radiographic findings. Radiation necrosis was defined based on radiographic criteria previously described [13]. Briefly, 3 criteria were assessed to determine the likelihood of RN: 1) increased T1 enhancement within the high dose region associated with increased peripheral edema and a central region of hypo-intensity, 2) a decrease or resolution of enhancement on subsequent follow up imaging, and 3) absence of increased vascular flow on perfusion-weighted MRI sequences. Adverse events (AEs) were graded based on Common Terminology for Adverse Events (CTCAE) version 5. AEs occurring in the presence of local or distant CNS progression were excluded.

### Statistical analysis

Baseline characteristics of lesions treated with SF-SRS and HF-SRS were compared using Wilcoxon rank sum test for continuous variables and Chi-square test for categorical variables. Tumor size (≤2 cm and > 2 cm) was analyzed as a categorical variable. The co-primary endpoints, local control (LC) and incidence of radiation necrosis (RN), were estimated using the Kaplan-Meier method and compared using the log-rank test. Time to local failure and RN were defined from the start date of SRS to the date of the event or to the date of last follow up for lesions without an event. Salvage WBRT for distant brain failure (LC analysis) or any brain failure (RN analysis) was considered a competing risk and used as a censoring event. Overall survival (OS) and distant brain failure (DBF) were estimated using the Kaplan-Meier method from the time of first SRS treatment to the date of event or date of last follow up. Cox proportional hazards model was used for univariate and multivariate analyses (MVA) to evaluate the effects of tumor/treatment characteristics on the clinical outcomes of interest. Propensity score (PS) adjustments were performed using inverse probability of multiple-fraction weighting. The PS included adjustments for age, gender, race, tumor size, histology, surgical resection, prior WBRT, and concurrent systemic therapy. The statistical program used was SAS (version 9.4, SAS Institute, Cary, NC). All statistical analyses were performed at a significance level of 0.05.

## Results

### Patient and tumor characteristics

One hundred and fifty six consecutive patients with a total of 335 metastatic brain lesions treated from 2013 to 2018 with SF-SRS (*n* = 222 lesions) or HF-SRS (*n* = 113 lesions) across 5 radiation centers were included. A summary of the patient characteristics at the time of first SRS treatment are shown in supplement Table 1. The most common primary site was lung and the median number of lesions treated per patient was 2 (range 1–15). A summary of tumor and treatment characteristics are shown in Table 1. The median tumor size was 0.7 cm (range 0.2–3.3 cm) for SF-SRS and 1.6 cm (range 0.2–5.0 cm) for HF-SRS. Lesions treated with SF-SRS were more likely to have a tumor size ≤2 cm (90%) compared to those treated with HF-SRS (63%) (*p* = 0.01). Prior WBRT was delivered to 33% (*n* = 74) and 34% (*n* = 38) of the lesions treated with SF- and HF-SRS, respectively (*p* = 0.72). The median dose of SF-SRS was 24Gy and the median BED_10_ of HF-SRS was 42.6 Gy.

**Table 1:** Tumor and treatment characteristics

	SF-SRS [n = 222 (%)]	HF-SRS [n = 113 (%)]	Unadjusted *p*-value	Adjusted *p*-value
Tumor location			0.64	0.82
Supratentorial	172 (78)	82 (73)		
Infratentorial	50 (22)	31 (27)		
Tumor size			< 0.001	0.01
≤ 2 cm	199 (90)	71 (63)		
> 2 cm	23 (10)	42 (37)		
Histology			0.07	0.07
Renal Cell	23 (10)	5 (4)		
Melanoma	18 (8)	14 (12)		
Squamous Cell	6 (3)	12 (11)		
Adenocarcinoma^a^	148 (67)	60 (53)		
Other	27 (12)	22 (20)		
Surgical resection			0.005	0.02
	8 (4)	18 (16)		
Prior WBRT			0.61	0.30
	74 (33)	38 (34)		
Concurrent systemic therapy			0.02	0.72
	73 (33)	18 (16)		

*WBRT* whole brain radiation therapy

^a^Adenocarcinoma includes adenocarcinoma of the lung and invasive ductal carcinoma of the breast

### Clinical outcomes

**Fig. 1:**
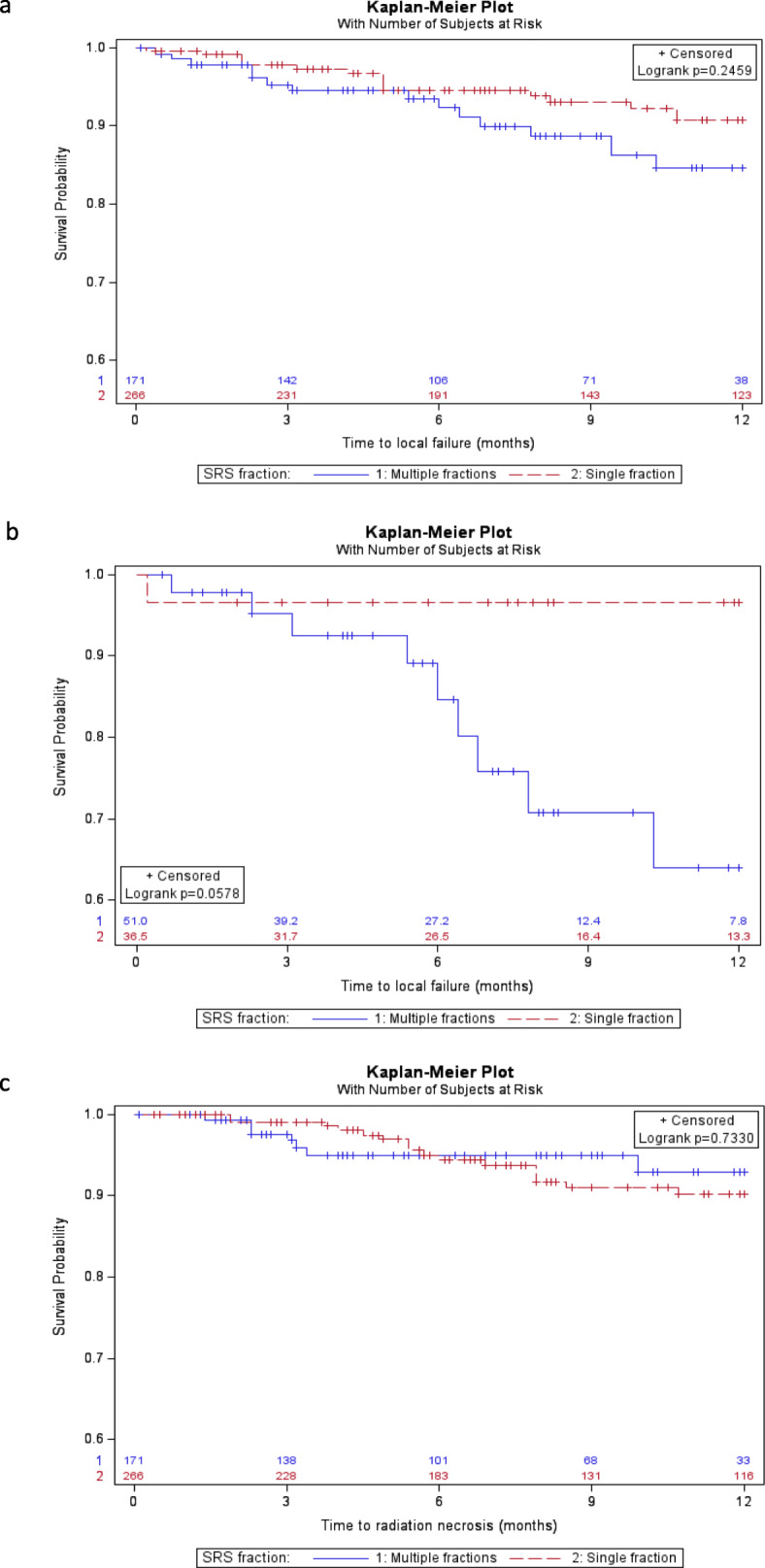
Kaplan Meier curves with propensity score adjustment showing (**a**) local control for the entire cohort, (**b**) local control for lesions > 2 cm and (**c**) radiation necrosis-free survival. SF-SRS (red/dotted) and HF-SRS (blue/solid)

The median follow up of each cohort was 12 months with an interquartile range (Q1-Q3) of 11.8–12 (HF-SRS) and 8–12 (SF-SRS) (*p* = 0.30). The one-year OS and DBF rate for the entire cohort was 46% (95% CI 38–54%) and 51% (95% CI 43–59%), respectively. The unadjusted 1 year LC was 90% (95% CI 85–94%) for SF-SRS and 81% for HF-SRS (95% CI 70–90%) (*p* = 0.04) (Suppl. Figure 1a); after PS adjustment the 1 year LC was 91% (95% CI 86–96%) and 85% (95% CI 75–95%) (*p* = 0.25), respectively (Fig. 1a). On univariate analyses tumor size predicted for increased risk of local failure, however, it was not significant on MVA (Table 2). On subgroup analysis of tumors > 2 cm, the unadjusted 1 year LC was 63% (95% CI 42–84%) and 96% (95% CI 81–99.7%) with HF-SRS and SF-SRS, respectively (*p* = 0.04) (Suppl. Figure 1b); after PS adjustment the 1 year LC was 64% (95% CI 43–85%) and 97% (95% CI 84–99.8%), respectively (*p* = 0.06) (Fig. 1b).

**Table 2:** Univariate and multivariate analysis for local failure

	Unadjusted HR (95% CI)	Unadjusted p-value	Adjusted HR (95% CI)	Adjusted *p*-value
Covariate				
Univariate				
Location of metastasis	1.07 (0.46, 2.50)	0.88	1.01 (0.47, 2.19)	0.98
Tumor size ≤2 cm	0.40 (0.19, 0.88)	0.02	0.44 (0.22, 0.88)	0.02
Histology^a^	1.26 (0.30, 5.30)	0.75	1.21 (0.31, 4.76)	0.78
Surgical resection	1.44 (0.44, 4.75)	0.54	1.42 (0.49, 4.12)	0.52
SF vs HF-SRS	2.10 (1.03, 4.33)	0.04	1.73 (0.90, 3.33)	0.10
Concurrent systemic therapy	0.79 (0.35, 1.78)	0.57	0.67 (0.31, 1.44)	0.30
Prior WBRT	1.97 (0.96, 4.04)	0.07	1.58 (0.82, 3.03)	0.17
Multivariate				
SF- vs HF-SRS	1.99 (0.84, 4.76)	0.12	1.65 (0.70, 3.87)	0.25
Tumor size ≤2 cm	0.49 (0.20, 1.17)	0.11	0.53 (0.23, 1.24)	0.14
Prior WBRT	2.10 (0.92, 4.81)	0.08	1.83 (0.77, 4.38)	0.17

^**a**^Histology was analyzed on UVA based on the 5 subgroups listed in Table 1 and was not statistically significant. To simplify, it is reported as a binary (squamous vs non-squamous) variable in this table

**Fig. 2:**
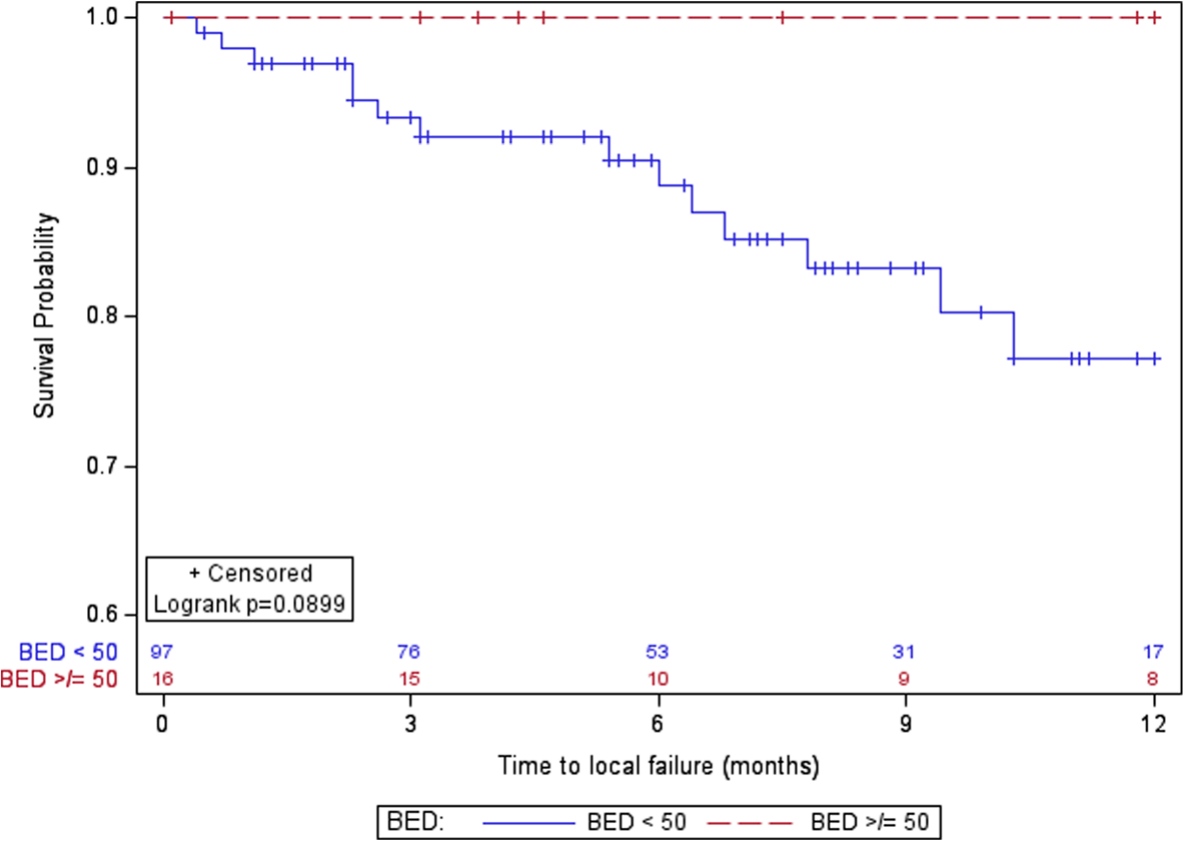
Kaplan Meier curve of local control for lesions treated with HF-SRS. BED_10_ < 50 (blue/solid) and BED_10_ ≥ 50 (red/dotted)

The HF-SRS cohort was further subdivided by BED_10_; One year LC of 100% for lesions treated with a BED_10_ ≥ 50 versus 77% (95% CI 65–88%) lesions for that received a BED_10_ < 50 (*p* = 0.09) (Fig. 2).

### Toxicity

Twenty-eight patients developed symptomatic RN (defined as grade 2 to 5 based on CTCAEv5). The incidence of symptomatic RN was not different between the two groups with an adjusted 1 year incidence of 10% (95% CI 5–15%) versus 7% (95% CI 0.1–14%) for SF-SRS and HF-SRS, respectively (*p* = 0.73), corresponding to a radiation-necrosis free survival of 90 and 93% (Fig. 1c). For HF-SRS, there was no difference in the incidence of RN between lesions treated with a BED_10_ ≥ 50 (0%) compared to a BED_10_ < 50 (11%; 95% CI 4–21%) (*p* = 0.27).

RN was diagnosed by T1/2-weighted MRI sequences (*n* = 28), MRI-perfusion study (*n* = 14) and pathology (*n* = 2). The most common presenting symptoms were headaches and/or motor weakness. The incidence of grade 2 (G2) and G3 RN was 8 and 11 (SF-SRS) and 5 and 2 (HF-SRS), respectively. There was one grade 4 toxicity after SF-SRS. One patient developed grade 5 toxicity 2 years after completing HF-SRS to 24Gy in 3 fractions after presenting with progressive right sided weakness, confusion and decreased functional status in the setting of steroid-refractory peri-lesional edema. While RN was considered most likely, local progression could not be completely ruled out. RN was treated with steroids (*n* = 27), Bevacizumab (*n* = 12) and surgical resection (*n* = 1). One patient with persistent grade 3 headaches resistant to steroids was treated with laser interstitial thermal therapy resulting in clinical improvement.

Tumor size ≤2 cm was associated with a significantly decreased risk of RN on univariate and multivariate analyses, which remained significant after PS adjustment (HR 0.21, 95% CI 0.07–0.58; *p* < 0.01) (Table 3). Neither SRS fractionation nor prior WBRT was associated with development of RN.

**Table 3:** Univariate and multivariate analysis for radiation necrosis

	Unadjusted HR (95% CI)	Unadjusted *p*-value	Adjusted HR (95% CI)	Adjusted *p*-value
Covariate				
Univariate				
Location of metastasis	0.94 (0.35, 2.56)	0.91	1.01 (0.41, 2.47)	0.98
Tumor size ≤2 cm	0.26 (0.11, 0.59)	0.001	0.25 (0.12, 0.54)	< 0.001
Histology^a^	1.70 (0.40, 7.25)	0.47	1.77 (0.45, 6.95)	0.41
Surgical resection	1.92 (0.57, 6.46)	0.29	1.71 (0.55, 5.35)	0.35
SF vs HF-SRS	1.07 (0.44, 2.61)	0.88	0.81 (0.36, 1.83)	0.61
Concurrent systemic therapy	0.55 (0.20, 1.48)	0.24	0.49 (0.19, 1.26)	0.14
Prior WBRT	1.72 (0.76, 3.90)	0.20	1.41 (0.66, 3.02)	0.37
Multivariate				
SF- vs HF-SRS	0.90 (0.28, 2.87)	0.86	0.96 (0.30, 3.03)	0.94
Tumor size ≤2 cm	0.20 (0.07, 0.57)	0.003	0.21 (0.07, 0.58)	0.003
Prior WBRT	1.35 (0.45, 4.03)	0.59	1.13 (0.35, 3.59)	0.84

^a^Histology was analyzed on UVA based on the 5 subgroups listed in Table 1 and was not statistically significant. To simplify, it is reported as a binary (squamous vs non-squamous) variable in this table

## Discussion

The aim of this study was to provide a real-world comparison of clinical outcomes between SF-SRS and HF-SRS in the treatment of brain metastases. Although, we found no difference in LC or RN between HF-SRS and SF-SRS cohorts, we observed a strong trend in improved LC with SF-SRS for larger lesions. Moreover, when further assessing HF-SRS regimens based on BED_10_, the LC appeared to improve when BED_10_ was > 50Gy, albeit not statistically significant.

The Radiation Therapy Oncology Group (RTOG) 90–05 prospectively established the standardized dosing regimen for SF-SRS after prior brain radiation. The maximum tolerated dose for tumors measuring 3.1-4 cm, 2.1-3 cm and ≤ 2 cm were identified as 15 Gy, 18 Gy and 24 Gy, respectively. The 2 year incidence of radiation necrosis using this approach was 11% and was directly correlated with tumor size. Since then, several institutional experiences have supported the efficacy and safety of single fraction radiosurgery in the treatment of small brain metastases [12, 13, 34–36]. However, studies have consistently shown an increased risk of radiation necrosis as well as decreased local control when treating larger tumors to a lower single fraction dose [2, 12–15].

The maximum tolerated dose of normal brain tissue is a major limitation of SF-SRS. Reduced setup margins and sub-millimeter dose distribution accuracy are necessary to reduce the dose to normal brain tissue as much as possible. Historically, this was only feasible using a single stereotactic coordinate system using a rigid frame attached to the patient’s skull. More recently, many centers are now utilizing a linac-based radiosurgery platform with real-time image guidance and a robotic couch capable of 6 degrees of rotational freedom. This technology provides the sub millimeter precision and accuracy of radiosurgery without the need for a surgically placed head frame. While the treatment approach is logistically appealing, access to this technology is limited due to the initial cost and additional resources needed to provide adequate quality assurance [25–27].

HF-SRS has been utilized, particularly when treating larger lesions, due concerns for increased toxicity and decreased tumor control with SF-SRS [16–20, 22, 23]. An Italian retrospective study compared the use of SF-SRS and HF-SRS in definitive treatment of brain metastases > 2 cm in size [29]. Lesions treated with HF-SRS received 27 Gy in 3 fractions and single-fraction dosing was 18Gy (lesions 2-3 cm) or 15–16 Gy (lesions ≥3 cm). At 1 year, they reported a significant improvement in local control for lesions treated with HF-SRS compared to those treated with SF-SRS which remained significant for larger (≥ 3 cm) tumors. Symptomatic RN was also significantly reduced with HF-SRS compared to SF-SRS. In contrast, despite that the majority of tumors in our study were ≤ 2 cm, we did not see a difference in LC or RN rates after PS adjustments. On a subset analysis of large tumors (> 2 cm), the unadjusted LC was significantly improved with SF-SRS and continued to trend in favor of SF-SRS after PS adjustments were made. We hypothesized these findings may be attributable to the wide range of HF-SRS regimens utilized in our study. Assuming an α/β of 10 for brain metastases using the linear quadratic model, local control was improved when the BED_10_ of the HF-SRS regimen was ≥50 Gy. Although this finding did not reach statistical significance, it was likely limited by sample size and warrants further prospective investigation.

A similar study from Germany compared the outcomes of 260 patients treated with either SF-SRS or two different HF-SRS regimens (5Gy × 7 or 4Gy × 10) [28]. The median PTV volumes were 0.87cm^3^, 2.04cm^3^ and 5.93cm^3^ for the SF-SRS, HF-SRS (7 × 5Gy) and HF-SRS (10 × 4 Gy) groups, respectively. There was no difference in local failure between the three cohorts, ranging from 8 to 11%. The two fractionation regimens utilized in this study had a BED_10_ ≥ 50 (52.5 for 5Gy × 7 and 56 for 4Gy × 10), which supports our observation of more durable local control with higher BED. Similarly, Chon et al. recently reported a SF-SRS versus HF-SRS comparison study limited to larger lesions measuring 2.5–3.0 cm which revealed significant improvement in local control and decreased risk of radiation necrosis associated with HF-SRS [37]. The median BED_10_ in their HF-SRS cohort was 59.5Gy compared to 42.6Gy in the present study.

A literature review of patients treated with HF-SRS using various dosing regimens is reported in Table 4 [16–23, 37–42]. While most studies report excellent 1 year local control with low rates of radiation necrosis (< 10%) associated with HF-SRS, there appears to be a dose response when the BED_10_ falls below 50Gy. The University of Alabama observed a significant increase in local failure associated with a 5 fraction regimen to a total tumor dose of 30Gy as compared with 25Gy corresponding to a BED_10_ of 48Gy and 37.5Gy, respectively [40]. Likewise, a Korean study of HF-SRS for large brain metastases showed total prescription dose ≥35 Gy was significantly correlated with local control on multivariate analysis [18]. There are several ongoing studies evaluating the optimal HF-SRS regimen for larger tumors/resection cavities (NCT01705548) and a randomized comparison of SF- vs HS-SRS in the post-operative setting (ALLIANCE A071801). However, until prospective data is available, we recommend aiming for a BED_10_ ≥ 50Gy when utilizing HF-SRS if normal tissue constraints can be met.

**Table 4:** Literature review of retrospective studies evaluating multi-fraction stereotactic radiosurgery

	# patients; # lesions	Median f/u (mos)	Tumor size (cm^3^)	Median dose (BED_10_)	1-year LC	Symptomatic RN	Prior WBRT	Prior resection
Median BED > 50								
Minniti et al. [24]	135;171	11.4	10.1	9Gy × 3 (51.3) (≥2 cm) 12Gy × 3 (79.2) (< 2 cm)	88%	6%	0	0
Aoyama et al. [38]	87; 159	6.3	3.3	8.75 × 4^a^ (65.6)	81%	2.7%	0	0
Ernst-Stecken et al. [16]	51; 72	7	13	7Gy × 5 (59.5) (n = 22) 6Gy × 5 (w/ WBRT) (48) (n = 29)	76%	NS	NS	NS
Fahrig et al. [17]	150; 228	28	6.1	5Gy × 7 (52.5) (*n* = 63) 7Gy × 5 (59.5Gy) (*n* = 51) 4Gy ×10 (56) (*n* = 36)	96% 87% 85%	NS	0	13%
Ogura et al. [23]	39; 46	9.5	3.7	7Gy × 5 (59.5)	87%	2.5%	71%	0
Manning et al. [20]	32; 57	9.2	2.2	9Gy × 3^a^ (51.3)	91%	6%	NS	19%
Jeong et al. [18]	37; 38	10	17.6	35Gy in 3/5 fxs (59–76)	87%	16%	0	0
Inoue et al. [39]	78; 85	8	12.6	6.2Gy × 5 (50.2)	93%	2%	13%	0
Navarria et al. [22]	102; 102	14	16.3	9Gy ×3 (51.3) (n = 51) 8Gy × 4 (57.6) (n = 51)	96%	6%	0	0
Chon et al. [37]	38 lesions	14	NS	7Gy x5^a^	92%	5%	0	0
Jeon et al. 2019 [46]	45; 52	10.5	14.2	8Gy × 3 (43) (n = 19) 9Gy × 3 (51.3) (n = 19) 10Gy × 3(60) (n = 14)	77%/100%^b^	17.8%	0	0
Median BED <50Gy								
Marcrom et al. [40]	72; 182	5	2	5Gy × 5 (37.5) (*n* = 48 lesions) 6Gy × 5 (48) (*n* = 134 lesions)	75% 91%	6%	7%	0
Narayana et al. [41]	20 patients	NS	NS	6Gy × 5 (45)	70%	NS	0%	0
Kwon et al. [19]	27; 52	6.6	0.5	5Gy x 5^a^ (37.5Gy)	68%	5.8%	86%	0

*LC* local control, *RN* radiation necrosis, *WBRT* whole-brain radiation therapy, *NS* not specified

^a^Median dose regimen

^b^Daily treatment/every other day treatment

The inherent bias of a retrospective patient chart review is the primary limitation of this study. Most notably, lesions treated with SF-SRS were smaller in size. Despite these differences, the median size of lesions in both cohorts was small (< 2 cm) and PS adjustment and regional modeling was used to account for this and other confounders. While the variability in HF-SRS dosing was considered another limitation, this prompted a subset analysis of dose-response which is a unique aspect to our study. Variations in SF-SRS prescribing patterns (i.e. 50% IDL for GK and 80% IDL for linac-SRS) result in differences in plan heterogeneity which may impact risk of RN and LC, however, clinical outcomes using these two modalities appear to be similar. Thus we decided to group these modalities into one cohort which is also consistent with national randomized trial design (ALLIANCE A071801).

Additional limitations of our study include the possibility of under-ascertainment of treatment related toxicity due to the subjective bias in radiographically distinguishing between radiation necrosis and local progression. While several studies that have evaluated alternative imaging modalities to better distinguish radiation necrosis from tumor progression [43, 44], these remain investigational. Furthermore, the rates of local control and radiation necrosis after SRS for brain metastases may be slightly skewed given that most patients succumb to their systemic disease, however, as systemic therapies continue to improve, these endpoints will likely become more meaningful. Lastly, the alpha/beta ratio used in the calculation of BED, is dependent on tumor histology and is likely overestimated when using a higher dose per fraction [45]. Therefore, the assumption of an alpha/beta ratio of 10 (i.e. BED_10_) for all tumors is an oversimplification of the actual radiobiologic events but was utilized to provide a reproducible method of comparing HF-SRS dose regimens.

## Conclusions

Here, we report no difference in local control or radiation necrosis rates between HF-SRS compared to SF-SRS in the treatment of brain metastases. Tumor size was the strongest predictor for development of radiation necrosis, which is consistent with prior studies. Lastly, we found a trend toward an improvement in local control when using a BED_10_ ≥ 50Gy for HF-SRS. Further analysis is needed to validate these findings and to determine the most optimal HF-SRS dose. Until prospective data is available, we recommend a dose regimen with a BED_1 0_ ≥ 50 Gy when HF-SRS is being utilized.

## Supplementary information

**Additional file 1: Supplemental Figure 1**. Kaplan Meier curves (unadjusted) showing (a) local control for the entire cohort, (b) local control for lesions > 2 cm and (c) radiation necrosis-free survival. SF-SRS (red/dotted) and HF-SRS (blue/solid).

**Additional file 2: Supplemental Table 1**.

## Data Availability

The datasets generated and analyzed during the current study are available in the University of Maryland RedCap repository: https://redcap-secure.igs.umaryland.edu. The datasets are not publicly available due to institutional privacy protocol but are available from the corresponding author upon reasonable request.

## Abbreviations

SF-SRS: Single-fraction stereotactic radiosurgery
HF-SRS: Hypo-fractionated stereotactic radiosurgery
LC: Local control
RN: Radiation necrosis
MVA: Multi-variate analysis
PS: Propensity Score
BED: Biologic Effective Dose
WBRT: Whole-brain radiation therapy
MRI: Magnetic resonance imaging
CTV: Clinical target volume
GTV: Gross target volume
PTV: Planning target volume
CBCT: Cone-beam cat scan
AEs: Adverse events
DBF: Distant brain failure
CNS: Central nervous system
