# Supplementary material for: A multi-center analysis of single-fraction versus hypofractionated stereotactic radiosurgery for the treatment of brain metastasis
Source: Radiat Oncol. 2020 May 28;15:128. doi: 10.1186/s13014-020-01522-6 (PMC7257186; doi:10.1186/s13014-020-01522-6)
Supplement: Supplementary file 1 — Additional file 1: Supplemental Figure 1. Kaplan Meier curves (unadjusted) showing (a) local control for the entire cohort, (b) local control for lesions > 2 cm and (c) radiation necrosis-free survival. SF-SRS (red/dotted) and HF-SRS (blue/solid). [file 13014_2020_1522_MOESM1_ESM.docx]

Supplement material:

**Table 1. Patient Characteristics**

|  | N=156 |
| --- | --- |
| Median Age (range) |  |
|  | 60 (28-89) |
| Gender |  |
| Female | 85 (54%) |
| Male | 71 (46%) |
| Race |  |
| White | 110 (71%) |
| Other | 46 (29%) |
| KPS |  |
| 80-100 | 120 (77%) |
| 50-70 | 36 (23%) |
| Primary Site |  |
| Lung  Breast Carcinoma | 86 (55%)  25 (16%) |
| Other | 45 (29%) |
| Extracranial Disease |  |
| None | 34 (22%) |
| Primary site | 41 (26%) |
| Limited bone^b^ | 10 (6%) |
| Extensive | 71 (46%) |
| Lesions treated with SRS^a^ |  |
|  | 2 (1-15) |
