# Supplementary figures and images for: A multi-center analysis of single-fraction versus hypofractionated stereotactic radiosurgery for the treatment of brain metastasis
Source: Radiat Oncol. 2020 May 28;15:128. doi: 10.1186/s13014-020-01522-6 (PMC7257186; doi:10.1186/s13014-020-01522-6)

## Slide 1
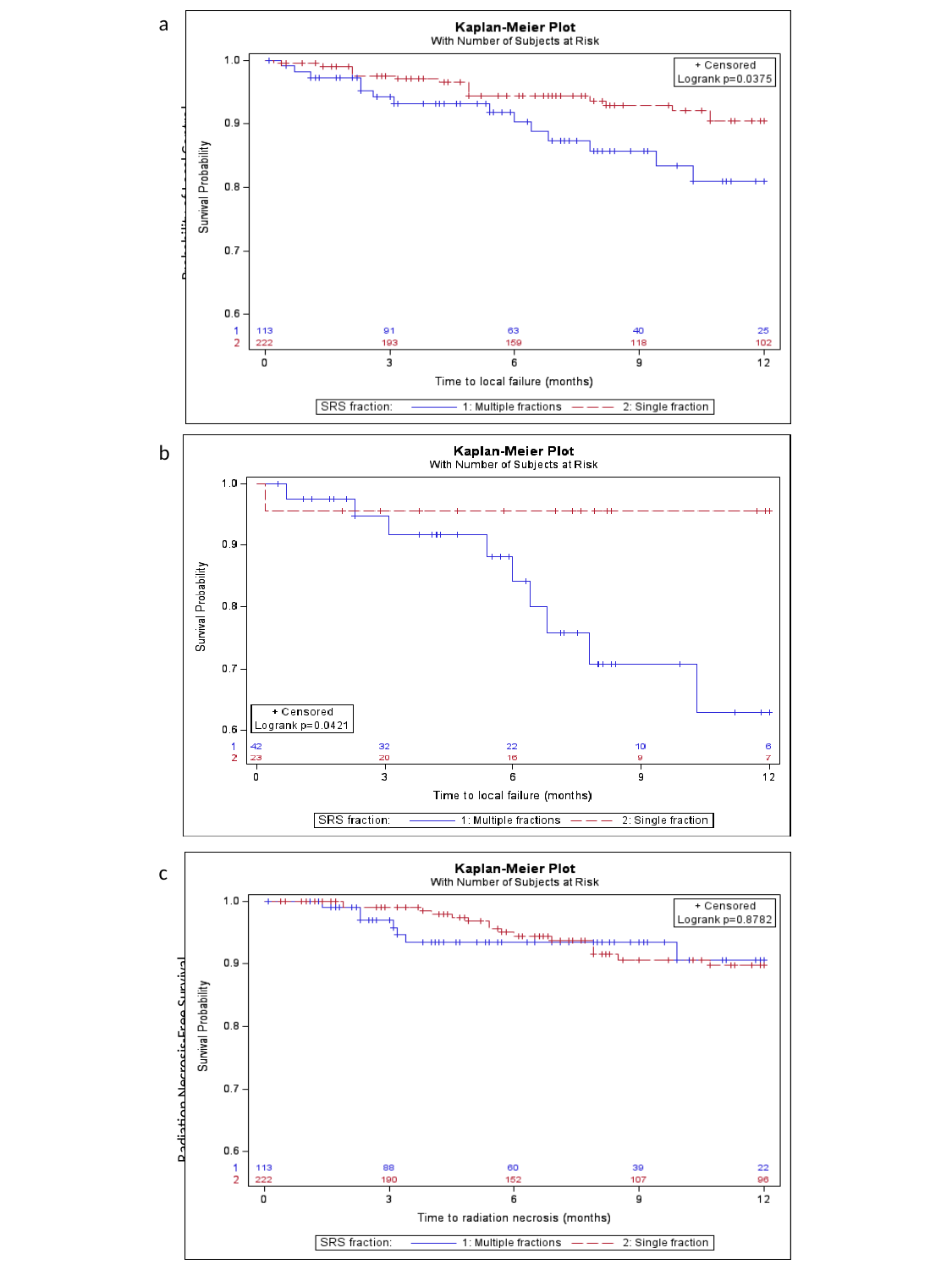

a
Probability of Local Control
Time (months)
b
c
Radiation Necrosis-Free Survival

Supplement: Supplementary file 2 — Additional file 2: Supplemental Table 1. [file 13014_2020_1522_MOESM2_ESM.pptx]
